# Induced hypothermia is protective in a rat model of pneumococcal pneumonia

**DOI:** 10.1186/cc9607

**Published:** 2011-03-11

**Authors:** C Beurskens, H Aslami, M Kuipers, M Schultz, N Juffermans

**Affiliations:** 1 Academic Medical Centre, Amsterdam, the Netherlands

## Introduction

Induced hypothermia is protective in ischemia-reperfusion injury by reducing the inflammatory response and is increasingly applied in the ICU. Hypothermia may dampen host response during an infection and it is believed that induced hypothermia may carry the risk of acquiring or aggravating an infection. We investigated the effect of hypothermia on bacterial outgrowth and on the inflammatory response in a rat model of pneumococcal pneumonia.

## Methods

Sprague-Dawley rats (350 to 400 g) were inoculated intratracheally with ~5.5 × 106 cfu of Streptococcus pneumonia, controls received saline. After 40 hours, the animals developed pneumonia and mechanical ventilation was started via a tracheotomy. Hypothermia (32°C) was induced using icepacks on the abdomen. In controls, normothermia was maintained by a thermomatrass. After 4 hours, rats were sacrificed, bronchoalveolar lavage fluid (BALF) was obtained and blood and organs were collected. Data are shown in percentages or median (range).

## Results

Induced hypothermia reduced pulmonary inflammation during pneumonia, exemplified by a reduction in pulmonary cell influx (1.3 (0.8 to 1.6) × 10^6 ^vs. 3.1 (1.6 to 4.6) × 10^6 ^mg/ml, hypothermia vs. normothermia; *P *< 0.05) and BALF protein levels (0.9 (0.6 to 1.3) vs. 1.5 (1.4 to 1.6) mg/ml; *P *< 0.05). Hypothermia also reduced BALF level of IL-1 (0.4 (0.1 to 0.6) vs. 0.8 (0.6 to 0.9) ng/ml; *P *< 0.05), but had no effect on BALF levels of CINC3 and IL-6. Hypothermia, however, did not affect bacterial outgrowth in the BALF (1.4 (0.3 to 20) vs. 0.5 (0.2 to 5.2) × 10^6 ^cfs/ml; *P *= NS) nor in homogenized lungs (13.5 (0.2 to 69.2) vs. 0.8 (0.1 to 14.5) × 10^6 ^cfu/g; *P *= NS). Hypothermia tended to reduce bacterial dissemination to the blood (38 vs. 50%, *P *= NS), spleen (0 vs. 50% culture positivity, *P *= 0.08) and liver (38 vs. 63% culture positivity, *P *= NS).

## Conclusions

Although hypothermia reduces pulmonary cell influx and protein leakage, it does not affect local bacterial outgrowth during pneumonia and even tends to reduce bacterial dissemination in this animal model of pneumococcal pneumonia. In contrast to current belief, induced hypothermia seems protective in a model of pneumococcal pneumonia.

